# Malignant Mixed Mullerian Tumor: An Immunohistochemical Study

**DOI:** 10.1155/2012/569609

**Published:** 2012-07-10

**Authors:** Zhanyong Bing, Theresa Pasha, Li-Ping Wang, Paul J. Zhang

**Affiliations:** Department of Pathology and Laboratory Medicine, Hospital of the University of Pennsylvania, Philadelphia, PA 19104, USA

## Abstract

Malignant mixed Mullerian tumor (MMMT) is an uncommon aggressive neoplasm composed of both malignant epithelial and mesenchymal components. In this study, immunohistochemical stains of germ cell markers, including SALL4, OCT3/4, glypican-3, and alpha-fetal protein (AFP), and CDX2 were performed in a series of MMMTs. SALL4 nuclear immunoreactivity was detected in 6 out of 19 cases (33%). The staining extent ranged from focal to extensive. The staining intensity was usually intermediate to strong (the score ranged from 1.5 to 3, and average score was 2.3 ± 0.5 in the positive cases). In addition, glypican-3 cytoplasmic reactivity was detected in 14 out of 16 cases (88%) with a mean score of 1.8 ± 0.7 (score ranging from 1 to 3). In contrast, OCT3/4 was only positive in 1 out of 19 cases and AFP in 2 out of 18 cases (11%). In summary, SALL4 and glypican-3 were frequently expressed in a subset of MMMTs. Their roles in the pathogenesis and biology of MMMT are yet to be determined. MMMT should be included in the differential diagnosis when a tumor is positive for SALL4 and/or glypican-3.

## 1. Introduction

Malignant mixed Mullerian tumor (MMMT) is an aggressive neoplasm that usually occurs in postmenopausal women and responds poorly to treatment [1]. Uterus and ovary are common sites for MMMT, though it can occur anywhere along the female genital tract and in the peritoneum. It is composed of both epithelial and mesenchymal components. The sarcomatous component can be homologous or heterologous depending on whether it is composed of native mesenchymal elements of the organ it arises from or other nonnative elements such as rhabdmyoblastic, osteogenic, chondroblastic, or lipoblastic element. The epithelial component can be endometrioid, undifferentiated, clear cell, or serous [1, 2]. The diagnosis of MMMT is usually not difficult; occasionally differentiation from various biphasic tumors such as mixed germ cell tumor can be difficult if the tumor occurred in relative younger age. In this study, we investigated the expression of germ cell markers including SALL4, OCT3/4, glypican-3, and AFP in a group of MMMTs. CDX2, a transcription factor regulating early enterogenic differentiation and found to express in some testicular germ cell tumor [3], was also evaluated in this group of MMMTs.

## 2. Material and Methods

Nineteen cases of MMMTs were retrieved from the database of the Hospital of the University of Pennsylvania, in which thirteen cases were from uterus, four from ovary, and two metastatic. One metastasis was from ovary to left paraaortic lymph nodes; the other was metastasized from uterus to rectal muscle. Immunohistochemistry was performed on 5 *μ*m formalin-fixed paraffin-embedded tissue sections. The antibodies used were listed in Table 1. All immunostains were performed on the fully automated Leica Microsystems' BondmaX Immunohistochemistry Staining System (Leica Microsystems, Bannockburn, IL). 

Sections were incubated with respective primary antibody for 15 minutes at room temperature followed by detection of the antigen with a biotin-free polymeric horseradish peroxidase (HRP)-linker antibody conjugate system (Refine Polymer, Leica Microsystems) for 8 minutes at room temperature. Blocking of endogenous peroxidase was performed by incubating tissue sections in 3.0% H_2_O_2_ for 7 minutes.

Immunoreactivity was evaluated semiquantitatively under light microscope. Immunoreactivities of SALL4, OCT3/4, glypican-3, AFP, and CDX 2 were scored according to their extent and intensity of immunoreactivity. The final scores were achieved by the sum of intensity (0, negative; 1, weak intensity; 2, moderate intensity; 3, strong intensity) and extent of the immunoreactivity (0, negative; 1, <30%; 2, 30–70%; 3, >70%) divided by 2.

## 3. Results

**Figure 1:**
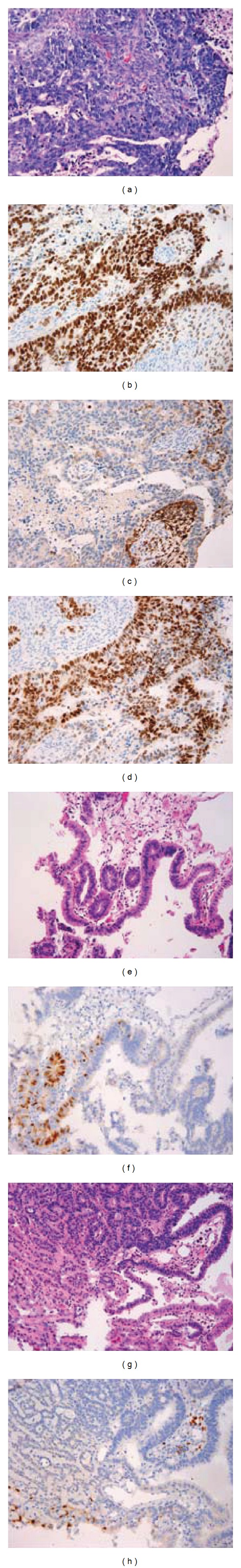
Malignant mixed Mullerian tumor. (a)–(d): one case of MMMT. (a) H&E, ×200; (b) SALL4, ×200; (c) glypican 3, ×200; (d) CDX2, ×200. (e)-(f) another case of MMMT. (e) H&E, ×200; (f) OCT3/4, ×200; (g) same case, different focus, H&E, ×200; (h) OCT3/4, ×200.

MMMTs were composed of both carcinomatous and sarcomatous components, which might show morphological similarity to malignant mixed germ cell tumor (Figure 1(a)). The immunohistochemical results for SALL4, OCT3/4, glypican-3, CDX2, and AFP were listed in Table 2 and as follows.

### 3.1. SALL4 

SALL4 nuclear stains were detected in 6 out of 18 cases (33%) with a score of 2.3 ± 0.5 in the positive ones. The extent of stain ranged from focal to diffuse, and the intensity of the stains was moderate to strong (Figure 1(b)). The positivity of SALL4 was usually detected in the epithelial components, which were usually high-grade serous adenocarcinoma. The mesenchymal component could show focal weak-to-moderate positivity. The benign endometrium, when present, was negative for SALL4 staining.

### 3.2. Glypican-3

Sixteen cases of MMMT were also stained for glypican-3, in which fourteen of them were positive (88%) for the cytoplasmic staining and the average score of stain was 1.8 ± 0.7 in the positive ones (Figure 1(c)). The extent of staining ranged from focal to diffuse, and intensity of the stains ranged from weak to strong. Glypican-3 reactivity was detected in both sarcomatous and poorly differentiated epithelial components. The benign endometrium, when present, was negative for glypican-3.

### 3.3. CDX2

Three out of 19 cases (16%) of MMMT were positive for CDX2 staining. The staining was in the nuclei of the epithelial components. The sarcomatous component was negative for CDX2. Two cases were focally positive, and the intensity of the stain was moderate (score 1.5). The remaining case showed diffuse and strong nuclear staining (score 3) (Figure 1(d)). Benign endometrium, when present, was negative for CDX2.

### 3.4. AFP

Two out of eighteen cases (11%) of MMMTs were also positive for AFP. One case was diffusely and the other case was focally positive. The intensities of the stain were moderate (score 2) (Figure 1(e)). The positive staining was in the epithelial component. Benign endometrium, when present, was negative for AFP.

### 3.5. OCT3/4

19 cases of MMMT were subject to OCT3/4 stain, and only one case showed focal nuclear positivity. The staining was mainly in the epithelial component (Figures 1(f) and 1(h)), which was a high-grade serous adenocarcinoma, while few mesenchymal cells were also positive (Figure 1(h)).

## 4. Discussion

MMMT is characterized by the presence of both carcinomatous and sarcomatous components with a poor prognosis [1]. Optimal cytoreduction seems to be the only therapy to have some impact on survival. No chemotherapy appears to show clear benefits [1]. MMMTs usually occur in postmenopausal women, however, may occasionally occur in relatively younger patients. Though most of germ cell tumors (GCTs) occur in younger patients, rarely can they occur in the postmenopausal women [4–6]. Some ovarian GCTs can sometimes be quite challenging in histological diagnosis. Mixed GCTs can mimic malignant mixed Mullerian tumors. YST can display multiple morphological patterns and can mimic different types of carcinoma such as clear cell carcinoma or endometrioid adenocarcinoma [7]. In difficult cases, various germ cell markers such as AFP and more recently described SALL4 and glypican-3 (GPC3) can be used to facilitate the diagnosis.

Recently, SALL4 has been reported to be positive not only in primitive germ cell tumors [8] but also in somatic malignancies [9, 10]. SALL4 is a zinc finger transcription factor and a homologue of drosophila spalt gene, which plays an important role in the specification of head and tail regions in the embryonic development [11]. In human, mutation of SALL4 causes the development of acro-renal-ocular and Okihiro syndromes [12]. SALL4 is important for the stem cell renewal and forms a regulatory circuit with OCT4, NANOG, and SOX2 to maintain embryonic stem cell pluripotency [13]. In addition to germ cell tumors, SALL4 has been shown to be expressed in somatic malignancy including lung [14], breast [9], leukemia [15] malignant rhabdoid tumor [16], and Wilm's tumor [17]. SALL4 appears to involve the proliferation and the self-renewal of cancer cells [10, 14]. It has been shown that SALL4 is only weakly positive in three out of 45 clear cell carcinomas in ovaries. All of other types of tumors from ovaries are negative for this marker [8]. In this study, we showed that SALL4 expression in MMMTs was significantly higher compared to the reported incidence in other ovarian epithelial tumors. Whether the higher expression of SALL4 in MMMT contributes to its more aggressive behavior needs further investigation.

The high percentage of SALL4 positivity in the MMMTs prompted us to study OCT3/4, a key regulator in the maintenance of pluripotency in stem cells [18]. OCT3/4 is a stem cell marker and has been used in the differential diagnosis of germ cell tumors [19]. OCT3/4 has been found to express in bladder cancer [20] and renal medullary carcinoma [21]. In the bladder cancer, OCT 3/4 expression was correlated with tumor grade and recurrence [20]. In addition, it was found that the positive tumor cells were distributed in clusters instead of a more diffuse pattern [22].

In this study, we found only one tumor to be focally positive for this marker. One possibility for such focal expression is that these cells are present very focally in the tumors and it is difficult to get a glimpse of them due to specimen sampling issue. 

Glypican-3 is a membrane-bound heparin sulfate proteoglycan. It is normally expressed in trophoblasts and a wide spectrum of fetal tissue and only limited expression in adult tissue [23]. It was first found to be overexpressed in YST by gene expression microarray [24]. Its expression was subsequently shown on routine histological section in YST [25]. Because of its selective expression in YST of mixed germ cell tumor, GPC3 can be used as a marker to differentiate YST among other germ cell elements. In this study, we showed that there was high percentage of positivity of GPC3 in both mesenchymal and epithelial components of MMMTs, indicating the stain for this marker has a limited role in the differential diagnosis between MMMT and YST. CDX2 is a homeobox domain containing transcription factor that is important for the development and differentiation of alimentary tract [26]. It has been shown to express in colonic epithelial tumors, and its role in tumorigenesis in colonic cancer has been speculated [27]. Despite the fact that CDX2 reactivity could be detected in small number of other non-GI tract epithelial tumors, it has been used as a diagnostic marker for tumor of GI tract origin in surgical pathology practice. We have demonstrated that variable CDX2 reactivity could be detected in the YST elements of mixed germ cell tumors with YST components in up to 40% of the cases and in mature colonic type epithelium in mature teratoma [3]. In this study, we showed low rate of positivity of this marker in MMMTs. In addition, we showed that two cases were also positive for AFP. AFP is a product of conceptus synthesized in yolk sac, liver, and GI tract in early fetal embryologic development. It is also characteristically expressed postnatally as fetal oncoprotein in YST and hepatocellular carcinoma as well as in some GI cancers such as gastric or colonic adenocarcinoma [28–30].

In summary, SALL4 was expressed in a subset of MMMTs with a higher frequency than that of other ovarian tumors. The mechanism of SALL4 in the pathogenesis and its role in the prognosis of this tumor need further investigation. In addition, MMMTs were also frequently expressing glypican-3 but rarely CDX2 and AFP. MMMT should be considered in the differential diagnosis when tumor is positive for both SALL4 and/or glypican-3. Both SALL4 and glypican-3 though considered as marker for germ cell tumor should not be used alone in differentiating MMMT from germ cell tumors.

## Figures and Tables

**Table 1:** Antibodies.

Antigen	Clone	Dilution	Antigen Retrieval	Manufacturer
SALL4	Mouse clone EE-30	1: 100	Citrate buffer, pH 6.0, 20 min at 100^°^C	Santa Cruz
OCT3/4	Goat polyclonal	1: 150	NO AR	Santa Cruz
Glypican-3	Mouse clone 1G12	1: 250	Citrate buffer, pH 6.0, 20 minutes at 100^°^C	Cell Marque
AFP	Rabbit polyclonal	1: 75	NA	Dako
CDX2	Mouse clone CDX2-88	1: 10	pH 9.0 EDTA, 20 min at 100^°^C	Biogenex

**Table 2:** Immunohistochemical stains in MMMTs.

	SALL4	OCT3/4	GPC3	CDX2	AFP
Number of cases	18	19	16	18	18
Number of positive cases	6	1	14	3	2
% of positivity	33	5	88	17	11
Score in positive cases	2.3 ± 0.5	1.5	1.8 ± 0.7	2	2
